# Caries-preventing effect of a hydroxyapatite-toothpaste in adults: a 18-month double-blinded randomized clinical trial

**DOI:** 10.3389/fpubh.2023.1199728

**Published:** 2023-07-18

**Authors:** Elzbieta Paszynska, Malgorzata Pawinska, Joachim Enax, Frederic Meyer, Erik Schulze zur Wiesche, Theodor W. May, Bennett T. Amaechi, Hardy Limeback, Amadeusz Hernik, Justyna Otulakowska-Skrzynska, Anna Krahel, Inga Kaminska, Joanna Lapinska-Antonczuk, Ewa Stokowska, Maria Gawriolek

**Affiliations:** ^1^Department of Integrated Dentistry, Poznan University of Medical Sciences, Poznan, Poland; ^2^Department of Integrated Dentistry, Medical University of Bialystok, Bialystok, Poland; ^3^Research Department, Dr. Kurt Wolff GmbH & Co. KG, Bielefeld, Germany; ^4^Society for Biometrics and Psychometrics, Bielefeld, Germany; ^5^Department of Comprehensive Dentistry, School of Dentistry, University of Texas Health San Antonio, San Antonio, TX, United States; ^6^Faculty of Dentistry, University of Toronto, Toronto, ON, Canada; ^7^Department of Gerostomatology, Medical University of Bialystok, Bialystok, Poland

**Keywords:** public health, adults, fluoride, toothpaste, randomized clinical trial (RCT), dental caries, Decayed Missing Filled Surfaces (DMFS) index, hydroxyapatite

## Abstract

**Background:**

Dental caries is a worldwide challenge for public health. The aim of this 18-month double-blinded, randomized, clinical trial was to compare the caries-preventing effect of a fluoride-free, hydroxyapatite toothpaste (test) and a toothpaste with sodium fluoride (1450 ppm fluoride; positive control) in adults.

**Methods:**

The primary endpoint was the percentage of subjects showing no increase in overall Decayed Missing Filled Surfaces (DMFS) index. The study was designed as non-inferiority trial. Non-inferiority was claimed if the upper limit of the exact one-sided 95% confidence interval for the difference of the primary endpoint DMFS between test and control toothpaste was less than the predefined margin of non-inferiority (Δ ≤ 20%).

**Results:**

In total, 189 adults were included in the intention-to-treat (ITT) analysis; 171 subjects finished the study per protocol (PP). According to the PP analysis, no increase in DMFS index was observed in 89.3% of subjects of the hydroxyapatite group and 87.4% of the subjects of the fluoride group. The hydroxyapatite toothpaste was not statistically inferior to a fluoride toothpaste with regard to the primary endpoint.

**Conclusion:**

Hydroxyapatite was proven to be a safe and efficient anticaries agent in oral care.

**Clinical trial registration:**

NCT04756557.

## 1. Introduction

Hydroxyapatite, Ca_5_(PO_4_)_3_(OH), is a calcium phosphate mineral that forms the mineral phase of human teeth and bone (1). Particulate biomimetic hydroxyapatite can be produced synthetically via different routes (2) and can be used for different purposes in medicine and dentistry (1, 3, 4). An important characteristic is its excellent biocompatibility and safety (3, 5). It is particularly useful in oral health as an active ingredient in oral care products, such as toothpastes (6–11), mouthwashes (12–16), and oral gels (17–19). Clinical studies have shown its efficacy in the improvement of periodontal health in patients with mild-to-moderate periodontitis (20), in the relief of dentin hypersensitivity (21, 22), and in other fields of application (23, 24).

The modes of action of hydroxyapatite in the oral cavity are based on physical, biochemical, and biological principles (23). Two key mechanisms involved in how toothpastes prevent dental caries are inhibiting demineralization and remineralization of initially demineralized tooth surfaces (25). In this respect, several *in vitro* and *in situ* studies that investigated hydroxyapatite toothpastes have been published (26–28). It has been shown that hydroxyapatite toothpastes remineralize enamel and dentin (8–10, 29) and inhibit demineralization (9, 19). Additionally, clinical studies have shown caries-preventing effects (6, 7, 26). Caries studies comparing hydroxyapatite and fluoride are important as fluoride has been used in oral care as an anticaries agent for decades (25, 30). However, it is known that chronic exposure of fluoride from various sources (drinking water, oral care products, food, etc.) has certain drawbacks. Fluoride can cause dental fluorosis and other side effects (31–35). As a consequence, the concentration of fluoride in toothpastes is limited and children up to 6 years are only allowed to use small amounts of toothpaste (pea size, grain of rice size) (36, 37). Reduced toothpaste amounts can lead, however, to a reduced cleaning efficacy compared with larger toothpaste amounts (i.e., full length of brush) (38).

The use of fluoride-free hydroxyapatite toothpastes has in recent years been shown to be a clinically proven approach to caries prevention (26). Paszynska et al. (6) showed the non-inferiority of a hydroxyapatite toothpaste to a toothpaste with 500 ppm fluoride (as amine fluoride) in children (6). Schlagenhauf et al. (7) have proven the non-inferiority of a toothpaste with hydroxyapatite to a toothpaste with 1400 ppm fluoride [as amine fluoride and stannous(-II-)fluoride] in adolescents undergoing orthodontic therapy (7). An important advantage of using hydroxyapatite in caries prevention is its pH buffering effect and its protective calcium- and phosphate-releasing properties in cariogenic biofilms (39).

The efficacy of hydroxyapatite-based toothpastes in preventing caries has been demonstrated in adolescents (7) and children (6) but not in adult populations. To fill this gap, the aim of this randomized clinical trial was to test the caries-preventing effect of a fluoride-free, hydroxyapatite toothpaste compared with a toothpaste with 1,450 ppm fluoride in adults. While the clinical studies from Paszynska et al. (6) and Schlagenhauf et al. (7) used commercial control toothpastes, the toothpastes formulations used in this study by the two study groups (test and control) just differ in the active ingredients, i.e., hydroxyapatite and fluoride. Thus, this caries study was aimed to directly compare hydroxyapatite and fluoride in toothpastes for daily oral care.

## 2. Materials and methods

### 2.1. Objectives

The study objective was the investigation of the caries-preventing effect in adults aged 18–45 regularly using a fluoride-free, hydroxyapatite toothpaste (test toothpaste) or the fluoridated control toothpaste. The aim of the study was to compare the caries preventive effect of the test and control toothpaste to prove the non-inferiority of the test toothpaste compared with the control toothpaste.

### 2.2. Study design

The study was performed as a two-centered, double-blinded, randomized, and active-controlled parallel-group study with two arms (control and test). The study was performed at two study centers, i.e., Universities of Medical Sciences, Poznan and Bialystok, Poland.

### 2.3. Inclusion and exclusion criteria

The following inclusion and exclusion criteria were applied:

A) Inclusion criteria

• Provision of written informed consent.
• Age 18-45 years (both men and women).
• A minimum of 10 caries-free (DMFS index=0) molars and premolars.
• Willing to use an electric (powered) toothbrush.

B) Exclusion criteria

*Medical reasons:*

• Untreated caries (subjects with untreated caries in need of restoration can become eligible after restorative therapy).
• Severe periodontitis at the baseline visit (pocket depth on at least one tooth ≥ 5.5 mm).
• Undergoing orthodontic treatment.
• Known allergy to one of the ingredients of the study toothpastes.
• Systemic disorders interfering with salivary function or flow rate.
• Regular medication intake interfering with salivary function or flow rate.

*Other reasons:*

• Participation in any other clinical study within the past 3 months or ongoing.
• Lack of intellectual or physical ability to conduct the study per protocol.
• Any other reason that, in the opinion of the investigator, disqualifies the subject from participating in the study.

### 2.4. Primary endpoint

Percentage of subjects showing no increase in overall Decayed Missing Filled Surfaces (DMFS) index (DMFS_Visit4_-DMFS_Visit1_ ≤ 0) during the observation period of 18 months (Visit 1: baseline visit; Visit 4: final visit after 18 months; for details see section 2.10).

### 2.5. Secondary endpoints

A) Percentage of subjects experiencing no change in mineral density (as analyzed by laser diode near-infrared light transillumination of dental tissues with the use of DIAGNOcam) during the observation period of 18 months.
B) Changes in the coverage of all teeth with bacterial plaque according to the criteria of the Plaque Control Record (PCR) during the observation period of 18 months (40).

### 2.6. Toothpastes (blinded)

The test toothpaste and the active control toothpaste were provided in neutral plastic tubes all having the same shape; thus, both the subjects and the investigators were blinded. It is pertinent to mention that the subjects were requested not to discuss anything related to the toothpastes with any of the study team member who was involved in the clinical examinations. The neutral plastic tubes were labeled with a random subject number for each subject. The toothpastes were produced by an external certified laboratory. The composition of the toothpastes for both groups was identical (except for the main active ingredients, i.e., hydroxyapatite and fluoride). The compositions of the study toothpastes were as follows.

#### 2.6.1. Test toothpaste—hydroxyapatite toothpaste (fluoride-free)

Aqua, hydrated silica, glycerin, hydrogenated starch hydrolysate, hydroxyapatite (10%), xylitol, silica, cellulose gum, sodium methyl cocoyl taurate, sodium sulfate, 1,2-hexanediol, aroma, caprylyl glycol, sodium cocoyl glycinate.

#### 2.6.2. Active control toothpaste—fluoride toothpaste

Aqua, hydrated silica, glycerin, hydrogenated starch hydrolysate, xylitol, silica, cellulose gum, sodium methyl cocoyl taurate, sodium sulfate, 1,2-hexanediol, aroma, caprylyl glycol, sodium fluoride (1,450 ppm fluoride), sodium cocoyl glycinate.

The composition of the hydroxyapatite test toothpaste was comparable to commercially available Karex toothpastes (Dr. Kurt Wolff GmbH & Co. KG, Bielefeld, Germany).

### 2.7. Application

Tooth brushing was performed twice a day (morning and evening; after the meals) for 3 min per episode. No other fluoride- and/or hydroxyapatite-containing oral care products, professional or home use, such as mouthwashes, gels, or fluoridate supplements, were allowed during the trial.

### 2.8. Toothbrushes

Electric (powered) toothbrushes (Oral-B; P&G, Schwalbach, Germany) were used by the subjects, and the brushing heads were changed every 2 months.

### 2.9. Clinical examinations

**A) Decayed Missing Filled Surfaces (DMFS) index**

DMFS Index calculation (41): There are five surfaces on the posterior teeth: facial, lingual, mesial, distal, and occlusal. There are four surfaces on anterior teeth: facial, lingual, mesial, and distal. The third molars were not counted.

• When a carious lesion or both a carious lesion and a restoration were present, the surface was listed as a D.

• When a tooth was extracted due to caries, it was listed as an M.

• When a permanent filling was present, or when a filling was defective but not decayed, this surface was counted as an F. Surfaces restored for reasons other than caries were not counted as an F. The total count was 128 surfaces for a maximum of 28 teeth.

**B) Examinations with DIAGNOcam**

DIAGNOcam was used according to the instructions of the manufacturer (KaVo Dental, Biberach, Germany). The following classification was used (42):

0 = Light transmission unchanged

1 = Shadow visible in enamel

2 = Shadow visible in dentin

**C) Plaque control record**

The Plaque Control Record (PCR) is an established method of recording the presence of the plaque on individual tooth surfaces (40). At the study visits, a plaque-disclosing solution (Gum Red-Cote, Sunstar Europe, Switzerland) was painted on tooth surfaces. After the subject had rinsed, the investigator (using an explorer or a tip of a probe) examined each stained surface for plaque accumulations at the dentogingival junction. After all teeth were examined and scored, the index was calculated by dividing the number of plaque-containing surfaces by the total number of available surfaces (Plaque Index Calculation = The number of plaque-containing surfaces/The total number of available surfaces).

### 2.10. Course of the study

**Visit 1: Study Day 0**

**Screening and baseline examination**

Potential subjects were informed by the investigator about the nature, significance, and scope of the clinical trial according to the requirements described in the written subject information. Before study enrollment, the willingness of the subjects to properly follow the study protocol throughout the 18 months of the study was assessed. Subjects were enrolled as study participants only when they had given their written informed consent. Subjects had to meet all inclusion criteria and no exclusion criteria. Subjects disqualified due to untreated caries became eligible after restorative therapy.

Once informed consent had been given, an initial examination took place that covered the following aspects:

- Screening subjects for study eligibility (inclusion and exclusion criteria).
- Collection of demographic data.
- Baseline data collection performed in the following sequence: PCR, DMFS, DIAGNOcam.

After the analysis of the PCR record using a plaque-staining solution, a professional tooth cleaning was performed. The professional tooth cleaning was performed at visits 1 and 4 only, and there was no fluoride application after the cleaning.

Finally, the study subjects received an electric toothbrush with three brushing heads (replaced every 2 months) and the allocated toothpaste (test or control) by a trained study nurse or dentist not involved in clinical study examinations.

The proper use of the assigned electric toothbrush and the issued toothpaste were also instructed by this study nurse or a dentist not involved in the clinical study examinations.

**Visit 2: Study day 182**

**1st follow-up examination**

One-hundred eighty-two days after the baseline visit, the PCR and DMFS were reassessed as described for the baseline visit. Subsequently, a study nurse or a dentist who has not involved in clinical study examinations handed out three new brushing heads for the electric toothbrush and a new supply of the assigned experimental toothpaste (test or control) for the next 182 days. Finally, subjects received a new appointment for visit 3.

**Visit 3: Study day 364**

**2nd follow-up examination**

Three hundred sixty-four days after the baseline visit, the procedure performed on Visit 2 was repeated, after which the subjects received a new appointment for Visit 4.

**Visit 4: Study day 546**

**Final visit**

Five hundred forty-six days after the baseline visit, the following assessments were repeated: PCR, DMFS after professional cleaning, and DIAGNOcam as described from the baseline visit.

An overview of the study course in detail is presented in Table 1.

**Table 1:** Study flowchart.

	**V 1**	**V 2**	**V 3**	**V 4**
	**Screening/baseline**			**Final visit**
**Timing**	**Study day 0 screening, collection of baseline data, study inclusion**	**Study day 182 1**^st^ **follow-up examination**	**Study day 364 2**^nd^ **follow-up examination**	**Study day 546 3**^rd^ **follow-up examination**
Informed consent procedure	x			
Review of inclusion and exclusion criteria	x			
Demographic data	x			
Randomization	x			
Dispense of five toothpaste tubes (test or control)	x	x	x	
DMFS	x	x	x	x
DIAGNOcam	x			x
PCR	x	x	x	x
Professional tooth cleaning	x			x
Dispense of electric toothbrush (plus three brushing heads)	x			
Dispense of three replacement brushing heads		x	x	
Instruction on proper use of electric toothbrush and issued toothpaste	x			
Efficacy check of oral hygiene efforts of subjects (if PCR is > 15%, training with subjects on efficacious brushing technique)		x	x	
Dispense of adverse event diary	x	x	x	
Return of adverse event diary and adverse event recording		x	x	x

### 2.11. Methods of determining safety

Safety assessments were performed in all subjects at every visit. The clinical examiner visually examined each subject's oral cavity and perioral area as well as questioned the subjects on any adverse events. All observed or voluntarily reported adverse events (AEs) and serious adverse events (SAEs) regardless of the experimental group were recorded.

### 2.12. Monitoring

The monitor (Dr. Egmont Zieseniß, Inpharm Consulting, Germany) reviewed the case report forms with the investigator and his staff at regular intervals:

1. First monitoring visit after 30% recruitment.
2. Second monitoring visit after 12 months.
3. Third monitoring visit at study close-out.

During the study, the monitor reviewed the case report forms at the above intervals to verify completeness, plausibility and consistency of the data, protocol adherence, and the progress of enrolment, and to ensure that study supplies are being stored, dispensed, and accounted for according to specifications.

### 2.13. Determination of sample size and statistical analysis

It was assumed that the primary endpoint (i.e., no increase in DMFS index, i.e., ΔDMFS = Visit 4 - Visit 1 ≤ 0) will occur in about 60% of study subjects. A sample size of 77 study subjects per arm (two arms) was calculated to be sufficient to reject the null hypothesis that the test toothpaste is inferior to the control toothpaste, using a non-inferiority margin of Δ = 20% for the primary endpoint one-sided chi-square test (α = 5%, power = 80%) as described in a previous study (7). SAS 9.4 (proc power two sample freq test = PCHI) was used for the sample size calculation.

A non-inferiority testing was performed. The non-inferiority margin Δ was set to 20% between the groups. The primary endpoint was analyzed for the per-protocol (PP) population and, in addition, repeated for sensitivity reasons for the ITT population. The confidence interval approach was used to test non-inferiority (43). Non-inferiority was claimed if the lower limit of the one-sided 95% confidence for the difference between test and control toothpaste was less than Δ ≤ 20%. The exact one-sided and two-sided 95% confidence intervals were calculated according to the method of Chan and Zhang (44) using SAS 9.4. In addition, a logistic regression analysis was performed with the primary endpoint as a dependent variable and toothpaste, center, gender, and age as independent variables (covariates).

The confidence interval approach was also performed to test non-inferiority of the secondary endpoint's percentage of subjects experiencing no increase in the overall number of caries lesions (as examined using DIAGNOcam) as explorative statistical evaluation. Moreover, a logistic regression analysis was performed for this secondary endpoint as a dependent variable and toothpaste, center, gender, and age as independent variables (covariates).

A two-sided independent *t*-test was applied for the secondary endpoint ΔPCR (=PCR Visit 4–PCR Visit 1) to compare test vs. control toothpaste. In addition, an analysis of covariance (ANCOVA) was performed for the secondary endpoint PCR using toothpaste, center, gender, and age as independent variables (covariates).

### 2.14. Ethical committee and study registration

The study for both centers was approved by the ethical commission of the Poznan University of Medical Sciences, Poznan, Poland (No. 691/20; November 04, 2020). The study was registered at ClinicalTrials.gov: NCT04756557.

## 3. Results

### 3.1. Subjects

Overall, 194 subjects were included: 97 (50%) applied the hydroxyapatite toothpaste (test toothpaste) and 97 (50%) applied the fluoride toothpaste (control toothpaste) as randomized. The study was prematurely terminated in 20 (10.3%) subjects for several reasons (test toothpaste: *n* = 11 [11.3%], control toothpaste: *n* = 9 [9.3%]). However, none of the dropouts were recorded due to the appearance of new caries.

All subjects who performed at least one follow-up visit (V2, V3, or V4) were included in the intention-to-treat (ITT) population. The study was terminated in five subjects at visit V1 (baseline); i.e., these subjects were not included in the ITT population. In total, 189 (97.4%) subjects were included in the ITT population. Finally, 174 (89.7%) subjects completed visit V4 (final visit): hydroxyapatite toothpaste: *n* = 86 (49.4%); fluoride toothpaste: *n* = 88 (55.6%). From these, 171 (82.1%) subjects were included in the per-protocol (PP) population.

The primary endpoint was analyzed for the PP population and, in addition, repeated for sensitivity reasons for the ITT population.

### 3.2. Patient flowchart and analysis sets

**Figure 1:**
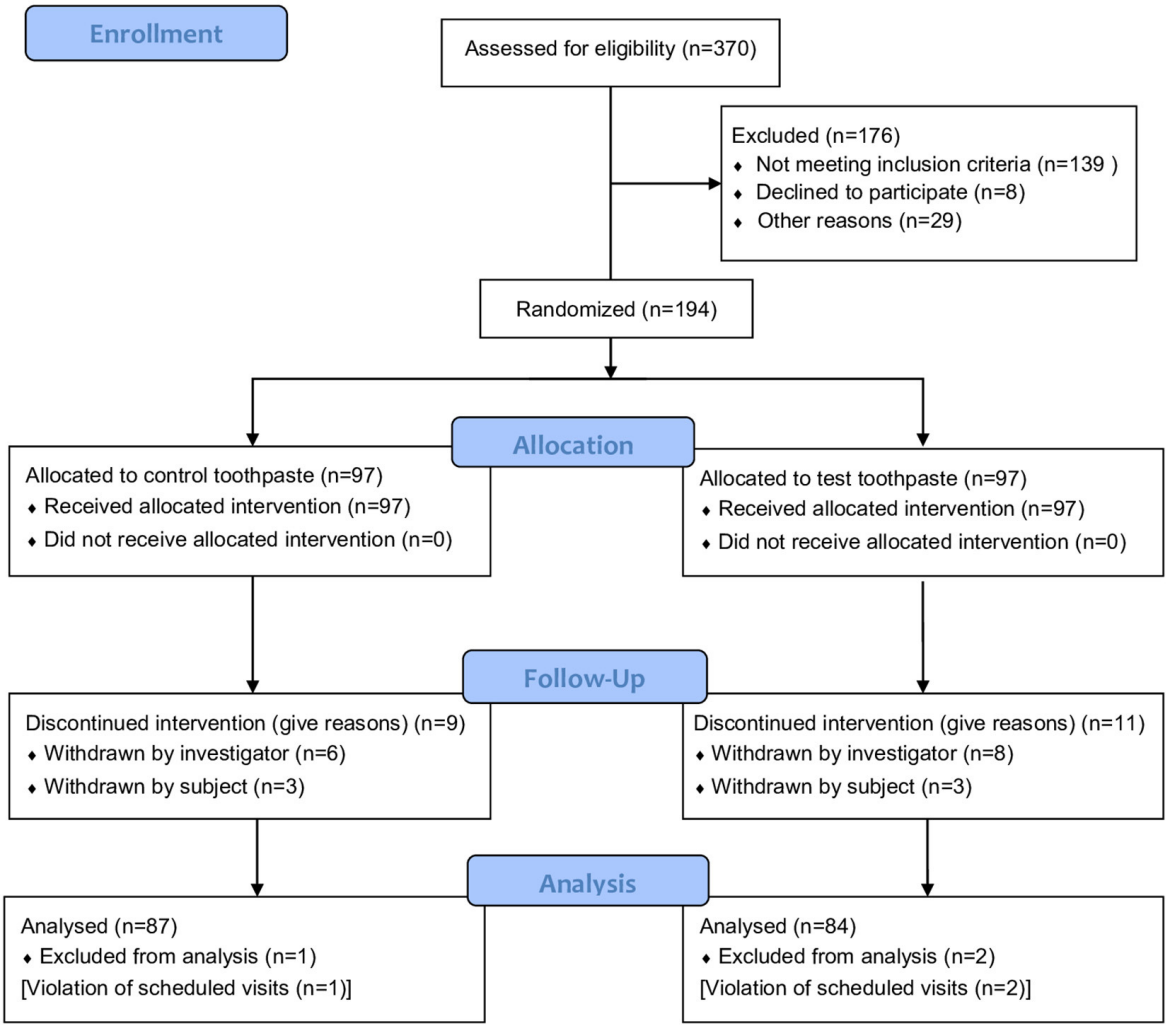
Patient flow chart.

The patient flow chart according to the CONSORT statement is shown in Figure 1.

In the following, the results are reported for the PP population (“analyzed subjects”) if not mentioned otherwise.

### 3.3. Demographic data

The demographic data of the PP population (*n* = 171) are summarized in Table 2.

**Table 2:** Summary of the demographic data of the PP population.

	**Control toothpaste**	**Test toothpaste**	**Total**
Female subjects	57 (65.5%)	57 (67.9%)	114
Male subjects	30 (34.5%)	27 (32.1%)	57
Age of the subjects in years: mean ± standard deviation	23.4 ± 3.7	23.3 ± 3.3	—

Age, weight, height, and sex did not differ significantly between groups (two-sided p-values > 0.10; t-test or chi-square test).

### 3.4. Primary efficacy parameter—DMFS index

#### 3.4.1. Descriptive statistics and figures

The descriptive statistics of the DMFS scores at visit V1 (baseline), visit V2 (6 months), visit V3 (12 months), and visit V4 (end of study, after 18 months) differentiated by toothpaste is shown in Table 3 for the PP population.

**Table 3:** Descriptive statistics of DMFS scores at visits V1, V2, V3, and V4 differentiated by group (control vs. test toothpaste); D+M+F, decayed missing filled surfaces.

**Group**		**Mean**	**SD**	**P25%**	**Median**	**P75%**	**Min**	**Max**	**N**
DMFS D (V1)	Control toothpaste	0.00	0.00	0.00	0.00	0.00	0.00	0.00	87
	Test toothpaste	0.00	0.00	0.00	0.00	0.00	0.00	0.00	84
DMFS M (V1)	Control toothpaste	0.11	0.75	0.00	0.00	0.00	0.00	5.00	87
	Test toothpaste	0.12	0.77	0.00	0.00	0.00	0.00	5.00	84
DMFS F (V1)	Control toothpaste	4.33	3.35	1.00	4.00	7.00	0.00	16.00	87
	Test toothpaste	3.77	3.04	1.00	4.00	5.00	0.00	15.00	84
DMFS D+F (V1)	Control toothpaste	4.33	3.35	1.00	4.00	7.00	0.00	16.00	87
	Test toothpaste	3.77	3.04	1.00	4.00	5.00	0.00	15.00	84
DMFS D+M+F (V1)	Control toothpaste	4.45	3.44	1.00	4.00	7.00	0.00	16.00	87
	Test toothpaste	3.89	3.17	1.00	4.00	5.50	0.00	15.00	84
DMFS D (V2)	Control toothpaste	0.02	0.21	0.00	0.00	0.00	0.00	2.00	87
	Test toothpaste	0.00	0.00	0.00	0.00	0.00	0.00	0.00	84
DMFS M (V2)	Control toothpaste	0.11	0.75	0.00	0.00	0.00	0.00	5.00	87
	Test toothpaste	0.12	0.77	0.00	0.00	0.00	0.00	5.00	84
DMFS F (V2)	Control toothpaste	4.38	3.32	2.00	4.00	7.00	0.00	16.00	87
	Test toothpaste	3.74	3.09	1.00	4.00	5.00	0.00	15.00	84
DMFS D+F (V2)	Control toothpaste	4.40	3.32	2.00	4.00	7.00	0.00	16.00	87
	Test toothpaste	3.74	3.09	1.00	4.00	5.00	0.00	15.00	84
DMFS D+M+F (V2)	Control toothpaste	4.52	3.44	2.00	4.00	7.00	0.00	16.00	87
	Test toothpaste	3.87	3.20	1.00	4.00	5.50	0.00	15.00	84
DMFS D (V3)	Control toothpaste	0.13	0.59	0.00	0.00	0.00	0.00	3.00	87
	Test toothpaste	0.00	0.00	0.00	0.00	0.00	0.00	0.00	84
DMFS M (V3)	Control toothpaste	0.11	0.75	0.00	0.00	0.00	0.00	5.00	87
	Test toothpaste	0.06	0.55	0.00	0.00	0.00	0.00	5.00	84
DMFS F (V3)	Control toothpaste	4.31	3.29	2.00	4.00	7.00	0.00	16.00	87
	Test toothpaste	3.69	3.09	1.00	4.00	5.00	0.00	15.00	84
DMFS D+F (V3)	Control toothpaste	4.44	3.35	2.00	4.00	7.00	0.00	16.00	87
	Test toothpaste	3.69	3.09	1.00	4.00	5.00	0.00	15.00	84
DMFS D+M+F (V3)	Control toothpaste	4.55	3.49	2.00	4.00	7.00	0.00	16.00	87
	Test toothpaste	3.81	3.22	1.00	4.00	5.50	0.00	15.00	84
DMFS D (V4)	Control toothpaste	0.05	0.26	0.00	0.00	0.00	0.00	2.00	87
	Test toothpaste	0.05	0.34	0.00	0.00	0.00	0.00	3.00	84
DMFS M (V4)	Control toothpaste	0.11	0.75	0.00	0.00	0.00	0.00	5.00	87
	Test toothpaste	0.12	0.77	0.00	0.00	0.00	0.00	5.00	84
DMFS F (V4)	Control toothpaste	4.57	3.35	2.00	4.00	7.00	0.00	16.00	87
	Test toothpaste	3.74	3.11	1.00	4.00	5.50	0.00	15.00	84
DMFS D+F (V4)	Control toothpaste	4.62	3.36	2.00	4.00	7.00	0.00	16.00	87
	Test toothpaste	3.79	3.11	1.00	4.00	6.00	0.00	15.00	84
DMFS D+M+F (V4)	Control toothpaste	4.76	3.47	2.00	4.00	7.00	0.00	16.00	87
	Test toothpaste	3.92	3.22	1.00	4.00	6.00	0.00	15.00	84

SD, standard deviation; P25%, 25% percentile; P75%, 75% percentile; Min, minimum; Max, maximum.

The descriptive statistics (Table 3) indicate that the mean DMFS index differed only slightly between groups (test toothpaste vs. control toothpaste) and changed only slightly in both groups from baseline (V1) to the end of study (V4).

The descriptive statistics of the differences in the DMFS Index (Delta V2 - V1, Delta V3 - V1, and Delta V4 - V1) are shown in Table 4 for the PP population.

**Table 4:** Descriptive statistics of differences of DMFS scores V2 - V1, V3 - V1, and V4 - V1 differentiated by group (control vs. test toothpaste).

**Group**		**Mean**	**SD**	**P25%**	**Median**	**P75%**	**Min**	**Max**	**N**
Delta DMFS V2 - V1	Control toothpaste	0.07	0.82	0.00	0.00	0.00	−2.00	7.00	87
	Test toothpaste	−0.02	0.84	0.00	0.00	0.00	−7.00	2.00	84
Delta DMFS V3 - V1	Control toothpaste	0.10	1.18	0.00	0.00	0.00	−6.00	7.00	87
	Test toothpaste	−0.08	1.27	0.00	0.00	0.00	−8.00	2.00	84
Delta DMFS V4 - V1	Control toothpaste	0.31	1.21	0.00	0.00	0.00	−2.00	7.00	87
	Test toothpaste	0.02	1.25	0.00	0.00	0.00	−7.00	3.00	84

SD, standard deviation; P25%, 25% percentile; P75%, 75% percentile; Min, minimum; Max, maximum.

**Figure 2:**
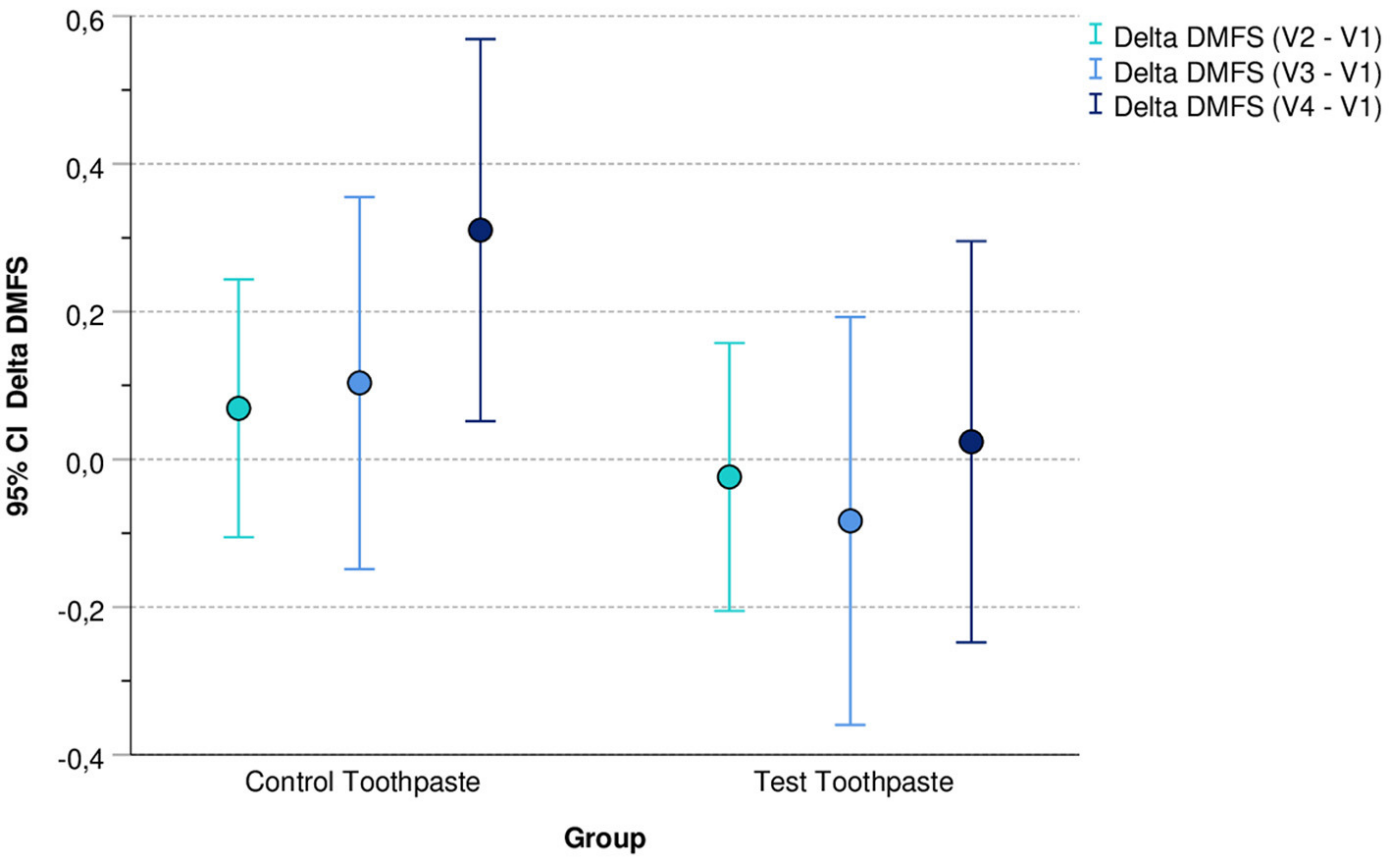
Mean values (and 95% confidence intervals) of differences between DMFS V2 - V1, DMFS V3 - V1, and DMFS V4 - V1 differentiated by group (control vs. test toothpaste).

Figure 2 illustrates the mean values and corresponding 95% confidence intervals (95% CI) of the differences between Delta DMFS V2 - V1, Delta DMFS V3 - V1, and Delta DMFS V4 - V1 differentiated by group (control vs. test toothpaste).

For the test toothpaste, the 95% confidence intervals of the differences include the value 0. This indicates that in this group the DMFS index did not change (statistically significant) during the application period compared with baseline. In contrast, in the group “Control Toothpaste” the 95% confidence interval for the difference DMFS V4 – V1 did not include the value 0 (DMFS V4 – V1 > 0). This indicates a (statistically significant) increase in DMFS in this group.

**Figure 3:**
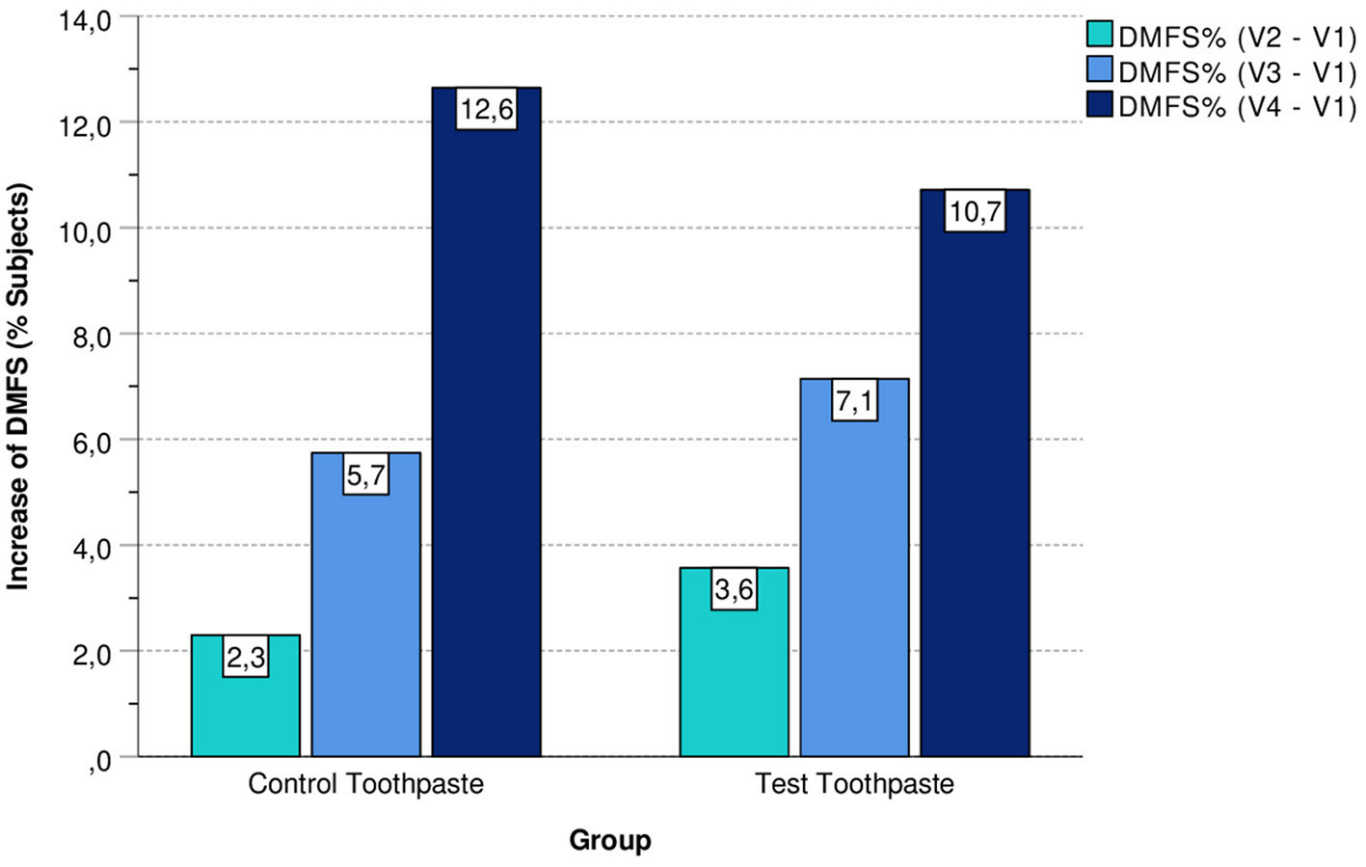
Subjects (%) with an increase in DMFS differentiated by group (control vs. test toothpaste).

In addition, Figure 3 shows the percentage of subjects with an increase in DMFS index at visits V2, V3, and V4 compared with the baseline (V1) differentiated by group (control vs. test toothpaste).

#### 3.4.2. Confirmative statistical test (primary analysis) and additional statistical tests

The primary efficacy endpoint was the percentage of subjects showing no increase in DMFS Index (ΔDMFS = DMFS _Visit4_ - DMFS _Visit1_ ≤ 0) during the application period of 18 months (Visit 4 - Visit 1) (Table 5). For comparison of the efficacy of the test toothpaste and control toothpaste, the difference in the percentages of subjects with ΔDMFS ≤ 0 was calculated:

**Table 5:** Percentage of subjects without an increase of DMFS Index in the PP and ITT population.

		**Toothpaste**					
		**Control toothpaste**		**Test toothpaste**		**Total**	
		***n***	**% (col)**	***n***	**% (col)**	***n***	**% (col)**
ΔDMFS% (PP) DMFS _Visit4_ - DMFS _Visit1_	≤ 0 (no increase)	76	87.36%	75	89.29%	151	88.30%
	> 0 (increase)	11	12.64%	9	10.71%	20	11.70%
	Total	87	100%	84	100%	171	100%
ΔDMFS% (ITT) DMFS _Visit4_ - DMFS _Visit1_	≤ 0 (no increase)	84	88.42%	85	90.43%	169	89.42%
	> 0 (increase)	11	11.58%	9	9.57%	20	10.58%
	Total	95	100%	94	100%	189	100%

ΔDMFS%_CT_ = ΔDMFS%_C_ - ΔDMFS%_T_
With ΔDMFS%_C_ = percentage of subjects in the control group with ΔDMFS ≤ 0
ΔDMFS%_T_ = percentage of subjects in the test group with ΔDMFS ≤ 0.

The upper limit of the one-sided 95% confidence interval for the difference in percentage of subjects without an increase of DMFS (ΔDMFS%_CT_ = −1.93%) was 6.84% and clearly below the non-inferiority margin of Δ ≤ 20% in the PP analysis. Thus, the test toothpaste (hydroxyapatite) can be considered non-inferior to the control toothpaste (1,450 ppm fluoride). This was also true for the upper limit of the two-sided 95% confidence interval (8.24%). In the ITT population, the upper limit of the one-sided 95% confidence interval for the difference in percentage of subjects without an increase of DMFS (ΔDMFS%_CT_ = −2.00%) was 5.68% and also clearly below the non-inferiority margin of Δ ≤ 20%. This was also true for the upper limit of the two-sided 95% confidence interval (7.27%), i.e., the “sensitivity analysis” is in accordance with the PP analysis. Superiority cannot be assumed as the confidence intervals include zero. The two-sided 95% confidence intervals for ΔDMFS%_CT_ in the PP population (−12.2%, 8.24%) and ITT population (−11.4%, 7.27%) lay within the range of (−20%, +20%). Assuming a margin of equivalence for ΔDMFS%_CT_ of (−20% to +20%), this indicates equivalence of the test and control toothpaste.

In addition, a logistic regression analysis was performed with the primary endpoint (increase in DMFS vs. no increase in DMFS) as dependent variable and toothpaste, center, sex, and age as independent variables (covariates).

The results for the PP population confirmed that the “risk” of an increase in DMFS did not differ significantly between control and test toothpastes.

Furthermore, the risk of an increase in DMFS did not differ significantly between centers, was not dependent on age, but was significantly higher in men compared to women. The corresponding odds ratio revealed that the “risk” of an increase in DMFS was 4.7-fold higher in men: An increase in DMFS was observed in 13 of 57 (22.8%) men and only in 7 of 114 (6.1%) women of the PP population.

### 3.5. Secondary efficacy parameters

Secondary efficacy parameters were (A) the overall number of caries lesions and (B) the coverage of all teeth with bacterial plaque according to the criteria of the PCR index (40) during the application period of 18 months.

#### 3.5.1. NCL—Descriptive statistics and figures

The overall number of caries lesions was assessed by DIAGNOcam (Table 6).

**Table 6:** Descriptive statistics of NCL scores 0, 1, 2, and [1 + 2] at V1 and V4 differentiated by group.

**Group**		**Mean**	**SD**	**P25%**	**Median**	**P75%**	**Min**	**Max**	**N**
NCL [0] (V1)	Control toothpaste	119.0	5.1	116.0	120.0	122.0	101	127	87
	Test toothpaste	119.1	4.6	115.5	120.0	123.0	109	126	84
NCL [1] (V1)	Control toothpaste	7.5	4.0	5.0	7.0	10.0	1	17	87
	Test toothpaste	8.2	4.3	5.0	8.0	12.0	2	18	84
NCL [2] (V1)	Control toothpaste	1.1	2.0	0.0	0.0	2.0	0	11	87
	Test toothpaste	0.7	1.1	0.0	0.0	1.0	0	5	84
NCL (V1) NCL [1 + 2] (V1)	Control toothpaste	8.7	4.5	6.0	8.0	12.0	1	21	87
	Test toothpaste	8.9	4.6	5.0	8.0	12.0	2	19	84
NCL [0] (V4)	Control toothpaste	118.4	4.6	115.0	119.0	122.0	106	127	87
	Test toothpaste	118.4	4.6	115.0	118.0	122.0	109	126	84
NCL [1] (V4)	Control toothpaste	8.3	4.0	5.0	8.0	11.0	1	17	87
	Test toothpaste	8.8	4.2	5.0	9.0	12.0	2	18	84
NCL [2] (V4)	Control toothpaste	1.2	2.1	0.0	0.0	2.0	0	11	87
	Test toothpaste	0.7	1.1	0.0	0.0	1.0	0	4	84
NCL (V4) NCL [1 + 2] (V4)	Control toothpaste	9.5	4.5	6.0	9.0	13.0	1	21	87
	Test toothpaste	9.5	4.5	6.0	9.5	13.0	2	19	84

[0] = light transmission unchanged, [1] = shadow visible in enamel, [2] = shadow visible in dentin. NCL was not determined at V2 and V3. SD, standard deviation; P25%, 25% percentile; P75%, 75% percentile; Min, minimum; Max, maximum.

Apparently, the percentage of surfaces classified as “score 2” (shadow visible in dentin) was very low at baseline (V1) in the group “control toothpaste” (0.9%) and in the group “test toothpaste” (0.6%) and did not change significantly during the application period in either groups at V4 (0.9%, 0.5%, respectively), whereas the percentage of surfaces classified as “score 1” (shadow visible in enamel) increased slightly in the “control toothpaste” (5.9% vs. 6.5%) and “test toothpaste” (6.4% vs. 6.9%) groups. Please note that NCL was not determined at V2 and V3.

**Figure 4:**
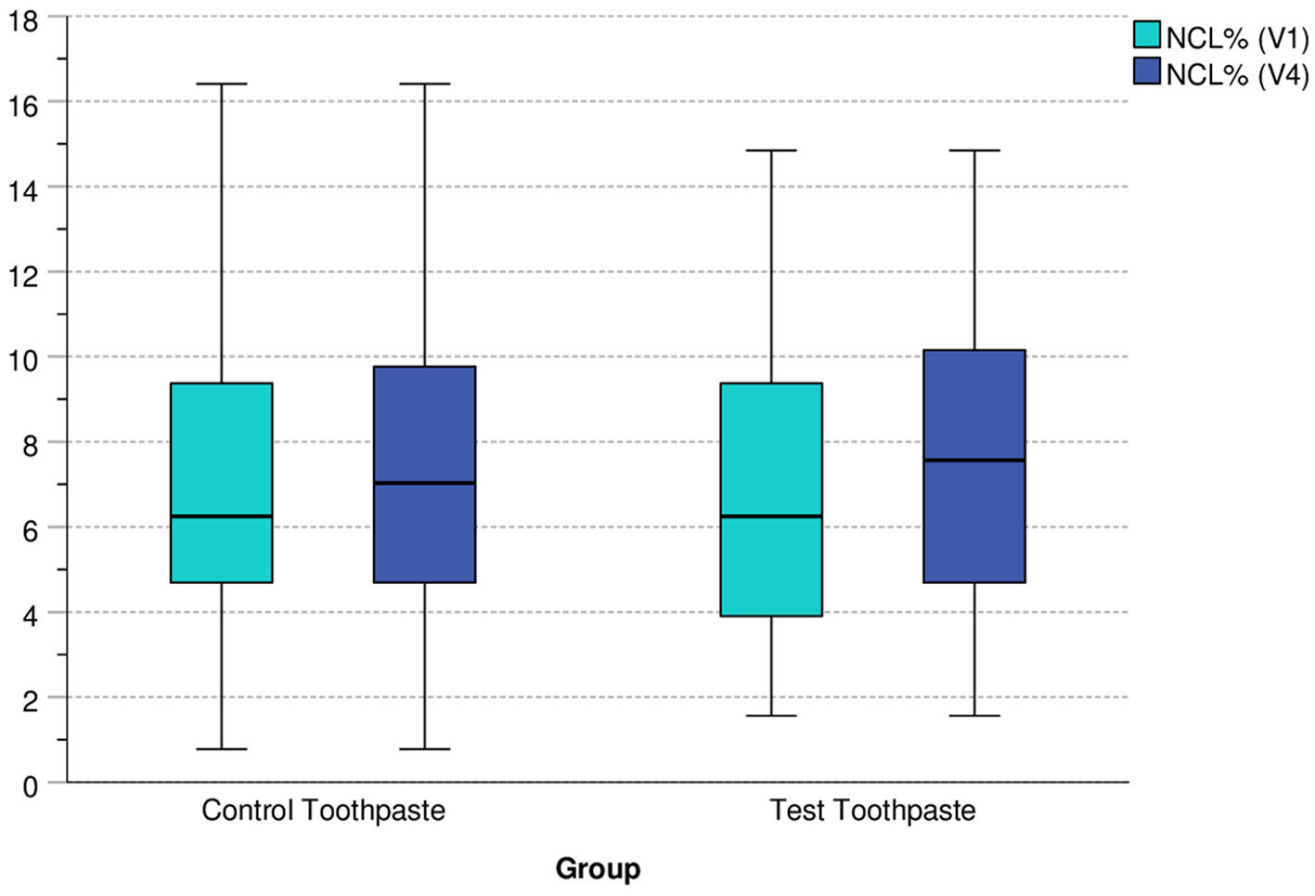
Boxplots of NCL% at V1 and V4 differentiated by group (control vs. test toothpaste).

Figure 4 indicates that the overall percentage of caries lesions NCL% differed only slightly between groups at V1 and V4 and increased only slightly between visit V1 (baseline) and V4 (after 18 months of applications of the toothpastes).

#### 3.5.2. NCL—explorative statistical tests

The secondary efficacy endpoint for NCL is the proportion of subjects showing no increase in NCL% (ΔNCL = NCL% _Visit4_ - NCL% _Visit1_ ≤ 0) during the application period of 18 months (Visit 4 - Visit 1) (Table 7). For comparison of efficacy of test toothpaste and the control paste, the difference of the percentages of subjects with ΔNCL ≤ 0 was calculated:

ΔNCL%_CT_ = ΔNCL%_C_ - ΔNCL%_T_
With ΔNCL%_C_ = percentage of subjects in the control group with ΔNCL ≤ 0
ΔNCL%_T_ = percentage of subjects in the test group with ΔNCL ≤ 0.

**Table 7:** Percentage of subjects without increase of NCL% (percentage of caries lesions) in the PP set.

		**Group**					
		**Control toothpaste**		**Test toothpaste**		**Total**	
		***n***	**% (col)**	***n***	**% (col)**	***n***	**% (col)**
ΔNCL% (PP) NCL% _Visit4_ - NCL _Visit1_	≤ 0 (no increase)	50	57.47%	51	60.71%	101	59.06%
	> 0 (increase)	37	42.53%	33	39.29%	70	40.94%
	Total	87	100.00%	84	100.00%	171	100.00%

The upper limit of the one-sided 95% confidence interval for the difference in proportion of subjects without an increase of NCL (ΔNCL%_CT_ = −3.24%) of 9.27% was clearly below the non-inferiority margin of Δ20% in the PP analysis. This is also true for the upper limit of the two-sided 95% confidence interval (11.60%). This indicates also for the secondary endpoint ΔNCL% that the test toothpaste is non-inferior to the control toothpaste.

In addition, a logistic regression analysis was performed for the secondary endpoint NCL (increase in NCL vs. no increase in NCL) as dependent variable and toothpaste, center, sex, and age as independent variables (covariates). The results for the PP population confirmed that the “risk” of increase in NCL did not differ significantly between control and test toothpaste. Furthermore, the risk did not differ significantly between centers and was not dependent on age and sex.

#### 3.5.3. PCR—descriptive statistics and figures

**Figure 5:**
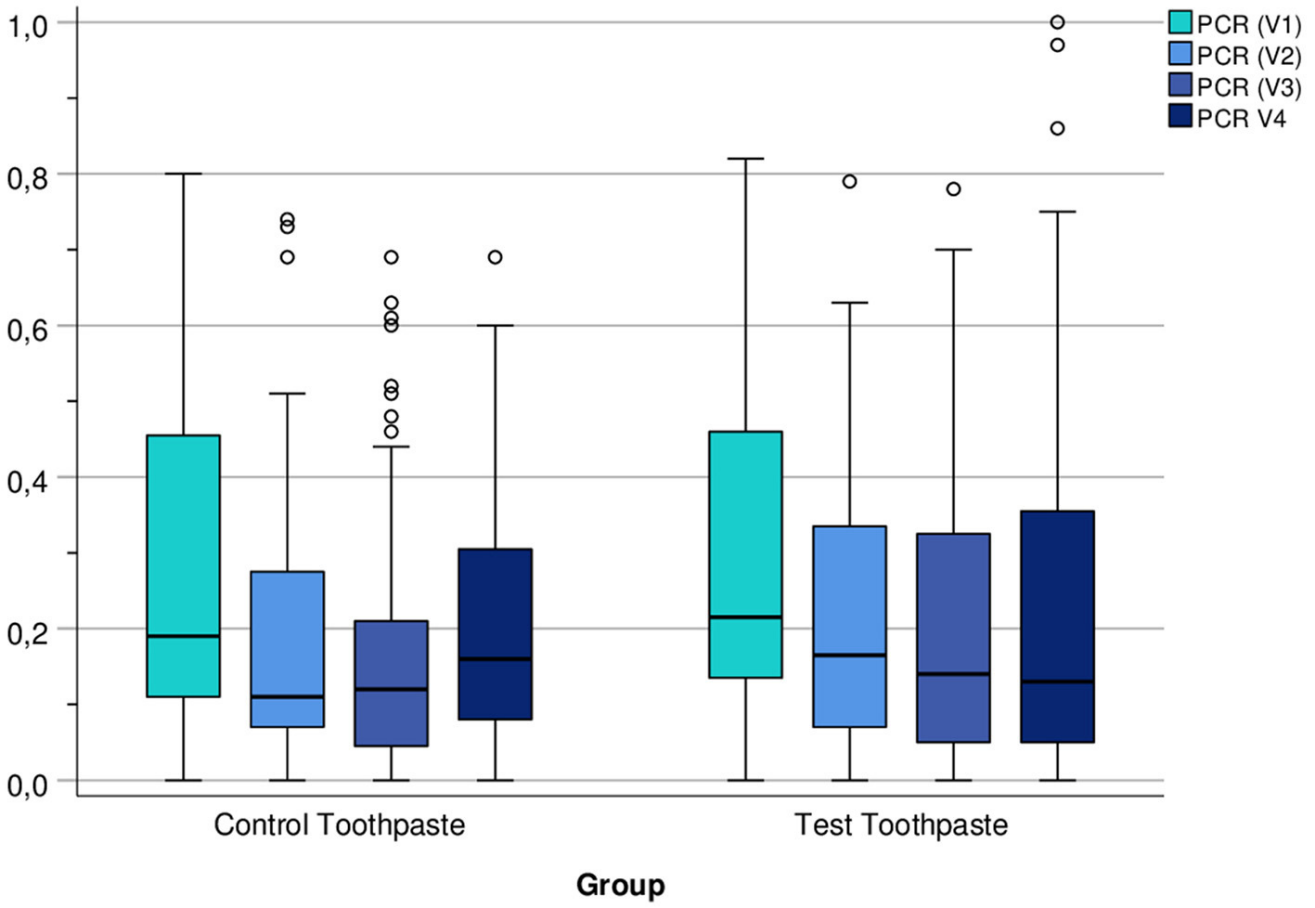
Boxplots of PCR Index (number of plaque-containing surfaces/total number of available surfaces) at visits V1, V2, V3, and V4 differentiated by group (control vs. test toothpaste).

The descriptive statistics (Tables 8–10) and Figure 5 indicate that the PCR scores differed only slightly between groups (test toothpaste vs. control toothpaste) and decreased slightly in both groups from baseline (V1) to the end of study (V4).

**Table 8:** Descriptive statistics of Plaque Control Record (PCR) at visits V1, V2, V3, and V4 differentiated by group.

**Group**		**Mean**	**SD**	**P25%**	**Median**	**P75%**	**Min**	**Max**	**N**
PCR (V1)	Control toothpaste	0.28	0.21	0.11	0.19	0.46	0.00	0.80	87
	Test toothpaste	0.30	0.22	0.14	0.22	0.46	0.00	0.82	84
PCR (V2)	Control toothpaste	0.18	0.17	0.07	0.11	0.28	0.00	0.74	87
	Test toothpaste	0.22	0.20	0.07	0.17	0.34	0.00	0.79	84
PCR (V3)	Control toothpaste	0.16	0.16	0.04	0.12	0.21	0.00	0.69	87
	Test toothpaste	0.21	0.20	0.05	0.14	0.32	0.00	0.78	84
PCR (V4)	Control toothpaste	0.20	0.17	0.08	0.16	0.32	0.00	0.69	87
	Test toothpaste	0.23	0.25	0.05	0.13	0.36	0.00	1.00	84

SD, standard deviation; P25%, 25% percentile; P75%, 75% percentile; Min, minimum; Max, maximum.

**Table 9:** Descriptive statistics of differences of Plaque Control Record ΔPCR (V2-V1), ΔPCR (V3-V1), and ΔPCR (V4-V1) differentiated by group (control vs. test toothpaste).

**Group**		**Mean**	**SD**	**P25%**	**Median**	**P75%**	**Min**	**Max**	**N**
ΔPCR (V2-V1)	Control toothpaste	−0.10	0.17	−0.13	−0.05	0.00	−0.66	0.44	87
	Test toothpaste	−0.08	0.15	−0.13	−0.06	0.01	−0.48	0.23	84
ΔPCR (V3-V1)	Control toothpaste	−0.11	0.17	−0.18	−0.08	0.00	−0.66	0.41	87
	Test toothpaste	−0.09	0.16	−0.18	−0.06	0.00	−0.57	0.33	84
ΔPCR (V4-V1) (ΔPCR)	Control toothpaste	−0.07	0.19	−0.18	−0.04	0.04	−0.58	0.33	87
	Test toothpaste	−0.07	0.21	−0.19	−0.06	0.04	−0.62	0.56	84

SD, standard deviation; P25%, 25% percentile; P75%, 75% percentile; Min, minimum; Max, maximum.

**Table 10:** Descriptive statistics for percentage changes in PCR (ΔPCR%) from baseline V1 to V2, V3, and V4 differentiated by group (PP).

**Group**		**Mean**	**SD**	**P25%**	**Median**	**P75%**	**Min**	**Max**	**N^a^**
ΔPCR% (V2-V1)/V1	Control toothpaste	−30.1	47.2	−56.9	**−36.4**	−3.3	−100.0	176.0	85
	Test toothpaste	−22.6	59.1	−58.7	**−25.0**	1.9	−100.0	200.0	83
ΔPCR% (V3-V1)/V1	Control toothpaste	−32.2	58.2	−77.8	**−38.9**	0.0	−100.0	205.0	86
	Test toothpaste	−25.1	84.8	−72.7	**−28.9**	0.0	−100.0	575.0	82
ΔPCR% (V4-V1)/V1	Control toothpaste	−7.3	79.4	−60.9	**−27.3**	20.7	−100.0	314.3	86
	Test toothpaste	−13.7	104.6	−69.2	**−32.5**	7.1	−100.0	650.0	83

^a^does not sum up to 171 because in single subjects (n = 2 / n = 3) PCR was 0 at V1 and > 0 at V2, V3, or V4, respectively. If PCR was 0 at V1 and 0 at V2, V3, or V4, respectively, the corresponding ΔPCR% was set to 0.

In addition, descriptive statistics for percentage changes in PCR from baseline to V2, V3, and V4 differentiated by group (control vs. test toothpaste) were calculated. Due to skewed distributions and extreme values, respectively, mean and standard deviation may be misleading, median, 25 and 75% percentiles should be interpreted instead. The medians indicate that percentage changes in PCR scores differed only slightly between groups (test toothpaste vs. control toothpaste), but decreased in both groups between V1 and V4, especially between V1 (baseline) and V2 (after 6 months) (Table 10).

#### 3.5.4. PCR—explorative statistical tests

A two-sided Mann–Whitney–Wilcoxon test was applied (instead of *t*-test) for the secondary endpoint ΔPCR to analyze the difference between test vs. control toothpaste, because Shapiro–Wilk test rejected normality (*p* = 0.003). Mann–Whitney test confirmed that ΔPCR (V4-V1) did not differ significantly between control and test toothpaste (two-sided *p* = 0.59). This was also true for PCR at V1 [baseline] (two-sided *p* = 0.65). The analysis of covariance of ΔPCR using toothpaste (control- vs. test toothpaste), center, sex, and age as independent variables (factors/covariates) was not performed, because Shapiro–Wilk test rejected normality. Additional Mann–Whitney tests did not indicate a significant effect of sex on ΔPCR (V4-V1). However, the decrease in ΔPCR was more pronounced in center Bialystok than in center Poznan (median −0.17 vs. −0.03). Additional Friedman tests indicate that PCR (V1, V2, V3, V4) decreased statistically significant in the control group and test group (*p* < 0.001 in each group).

### 3.6. Safety

All randomized subjects (*n* = 194), who received the control or test toothpaste at baseline (visit V1), were included in the Safety Analysis Set. No serious and no severe adverse events were reported. There were no statistically significant differences in causality and outcome of adverse events between the two toothpastes groups (*p* > 0.05, two-sided Fisher's exact test). In almost all AEs, the causality of AEs with the application of the toothpaste was rated as “unlikely” (control toothpaste: 99.0%, test toothpaste: 99.4%).

## 4. Discussion

In the present clinical trial, the effectiveness of hydroxyapatite toothpaste in preventing caries development was compared with that of fluoride (1,450 ppm) toothpaste, to determine the non-inferiority of the hydroxyapatite toothpaste to the fluoride toothpaste. Analysis of both the primary and the secondary outcome measures demonstrated non-inferiority of the two products to each other. This result is in agreement with two previously published clinical trials (6, 7); thus, this is the third clinical trial showing that fluoride-free hydroxyapatite toothpastes are non-inferior to fluoride toothpastes in caries prevention. Although not statistically significant in all three clinical trials, there was a tendency that the hydroxyapatite toothpastes were slightly more efficient than the corresponding fluoride control toothpastes.

The present study used a toothpaste with sodium fluoride (1,450 ppm fluoride) as control toothpaste. Sodium fluoride is one of the most frequently used fluoride compounds in fluoridated toothpastes worldwide (25). Walsh et al. (30) published a comprehensive systematic review on the use of fluoride toothpastes for cavity prevention (30). Even though most studies have been conducted and published more than 30 years ago (30, 45), for a comparability of those studies with recently published data, the methodology should at least be comparable. The authors defined, apart from fluoride toothpaste or concentration, the following inclusion criteria: The studies had to be randomized clinical trials with a study duration of at least 1 year. For caries detection, DMFS/T (Decayed Missing Filled Surfaces/Teeth) was used. These inclusion criteria are in line with the present study, in which the study duration was 18 months, and primary outcome was measured using DMFS, supplemented with a laser near-infrared transillumination by means of DIAGNOcam. Although a transillumination is considered a qualitative method of caries diagnostics, in the present study a classification was applied as published before (42), which enabled the quantitative assessment of caries lesions. The sample size of the present study was also comparable with those from the studies included in the review of Walsh et al. (30). Cardoso et al. (46) which is the most recent study included in the Walsh et al. (30) review, included approximately 50 subjects per group (46). The same sample size per group (*n* = 50) was included in Rao et al. (47). In general, sample size calculations were seldom provided by the other papers. Apart from this, no study included in Walsh et al. (30) compared a placebo toothpaste or any other fluoride toothpaste with a 1,450–1,500 ppm fluoride toothpaste in adults (30). Three studies compared toothpastes with 1,000 ppm fluoride to placebo toothpastes (48–50). All of those studies showed a significant caries prevention after 12 months of fluoride toothpaste use.

Approximately almost 90% of the subjects remained caries-free (no increase in DMFS) in the present study, representing the caries-preventing effect of both toothpastes within the study duration. This is higher than in data from Rao et al. (47): 31% in the placebo group, 53% in the fluoride group (1,190 ppm fluoride), and 72% in the casein phosphopeptide group remained caries-free after 2 years of using their respective toothpaste (47). However, this study was on children, which might be one reason for higher caries levels compared with the present study. For this reason, a direct comparison is not possible, e.g., due to different populations and time points of DMFS examination.

The primary parameter in the present study was the DMFS index. This index was established in clinical studies that investigated the caries-preventing effect of toothpastes (30). Based on the statistical analysis, a remineralizing effect was not excluded in the present study (DMFS _Visit4_ ≤ DMFS _Visit1_). According to the per-protocol analysis, no increase in DMFS index was observed in 89.29% of subjects of the hydroxyapatite group and 87.36% of subjects of the fluoride group. According to the intention-to-treat analysis, no increase in DMFS index was observed in 90.43% of the subjects of the hydroxyapatite group and 88.42% of the subjects of the fluoride group (Table 5). Assuming a margin of equivalence for the primary endpoint of (−20 to +20%), the results would indicate not only non-inferiority but equivalence of hydroxyapatite toothpaste and fluoride toothpaste.

When comparing the mean DMFS increase on the tooth surface level during the 18-month study duration with the previously published studies (48–50), the hydroxyapatite toothpaste showed the lowest increase (mean DMFS increase = 0.02). The performance of the fluoride toothpaste in this study (mean DMFS increase = 0.31) is between the efficacy of this measured by Lu et al. (49) (mean DMFS increase = 0.15) and Jensen and Kohout (48) (mean DMFS increase = 0.73). Based on the average DMFS from previously published studies, the results of this study are comparable with those from Muhler and Radike (50), Lu et al. (49), and Jensen and Kohout (48) (Table 11).

**Table 11:** Comparison on the mean increase in DMFS between this study and the published studies on adults using fluoridated toothpastes.

**Study**	**DMFS increase (fluoride group)**	**DMFS increase (hydroxyapatite group)**	**Study duration**	**Groups**
This study	0.31	0.02	1.5 years	NaF (1,450 ppm fluoride) vs. hydroxyapatite
**Study (publication year)**	**DMFS increase (fluoride group)**	**DMFS increase (placebo group)**	**Study duration**	**Groups**
Muhler et al. (50)	3.31	4.99	2 years	SnF_2_ (1,000 ppm fluoride) vs. placebo
Lu et al. (49)	0.15	0.88	1 year	SnF_2_ (1,000 ppm fluoride) with calcium pyrophosphate vs. placebo
Jensen et al. (48)	0.73	1.24	1 year	NaF (1,100 ppm fluoride) vs. placebo

In addition to DMFS, the overall number of caries lesions was determined with DIAGNOcam. DIAGNOcam is a modern device for laser diode near-infrared light transillumination of dental tissues. The appliance combines fiber optic transillumination with near-infrared (NIR) light and a digital camera to non-ionizing imaging of dental tissues. DIAGNOcam enables the visualization of caries lesions on occlusal and approximal surfaces and measures their severity in real time. It may be used without any limitation to monitor the caries process. It is a non-invasive and non-destructive tool (51). This novel infrared digital imaging transillumination technology shows a good accuracy and even a higher sensitivity and accuracy compared with bitewing radiographs in both *in vitro* (52–54) and *in vivo* studies (55, 56). The trend showing that the hydroxyapatite toothpaste is non-inferior to the fluoride toothpaste observed in the DMFS indices was clearly confirmed with DIAGNOcam (Tables 6, 7). According to the per-protocol analysis, no increase in percentage of caries lesions (DIAGNOcam) was observed in 60.71% of the subjects of the hydroxyapatite group and 57.47% of the subjects of the fluoride group (Table 7). It is pertinent to mention that in both study groups no subject was withdrawn for developing new caries.

The difference in DMFS (visual) and NCL based on DIAGNOcam (near-infrared light transillumination) can be explained by the sensitivity of this method. While decayed surfaces can only be detected on the outer area of the tooth visually by an experienced clinician, transillumination methods can also detect lesions underneath the surface (57, 58).

A notable trend was also observed in the PCR index of the hydroxyapatite group: Unlike the fluoride group, the PCR scores in the hydroxyapatite group showed a tendency to a lowering of scores during the course of the study (Figure 5). This is in line with previously published *in situ* studies on hydroxyapatite-based mouthwashes that showed that hydroxyapatite particles reduce the initial bacterial colonization on enamel surfaces (12, 13).

A strength of the present study is that the same toothpaste composition was used for the hydroxyapatite toothpaste and the fluoride toothpaste except that they differed in their main active ingredients (i.e., hydroxyapatite vs. fluoride). All other ingredients remained the same. While the non-inferiority of hydroxyapatite to fluoride toothpastes was shown in caries risk groups [children (6) and orthodontic patients (7)], this is the first study of this nature conducted in adults. Most studies on the caries-preventing effect of fluoride toothpastes were limited to children and adolescents (30). Additionally, the toothpastes were analyzed over 18 months in the present study which is a longer period than in previous anticaries studies on hydroxyapatite toothpastes (6, 7).

Secondly, as we tested the influence of both toothpastes on caries progression, the patients were not monitored according to dietary habits, and they kept their sugar intake under real-life conditions. There were no specific inclusion or exclusion criteria concerning the diet. However, the subjects had comparable diet behaviors due to living in urban areas in both study centers (59, 60). Recently published meta-analysis on sugar intake (including all mono- and disaccharides) in Eastern European countries showed high consumption of sweets, confectioneries, and drinks with high added sugar among the Polish adult population (59, 60). In addition, the amount of carbohydrates taken into the body through oral cavity has also deteriorated during the pandemic Covid-19 quarantine (61).

A limitation of this study is that the study population was relatively homogeneous, consisting of healthy, predominantly young adults without significant oral health problems. However, previously published RCTs on hydroxyapatite toothpastes were performed in caries risk groups, i.e., orthodontic patients (7) and children (6). A further limitation of the present study is that it was performed in a clinical setting with dental visits every 6 months; however, this is not the case for the whole population. Furthermore, no placebo toothpaste was used, but this is because it would be unethical to expose subjects to a risk of developing caries using a toothpaste without an anticaries agent. Additionally, based on evidence-based medicine, a placebo is only ethically reasonable, when there is no other known active treatment for the investigated disease. Another important note is that no bitewing radiographs were used to monitor caries progression. As studies have shown a better accuracy and sensitivity of DIAGNOcam compared to bitewing radiographs (57), there is no need to include diagnostic measurements based on X-rays and expose the subjects to a possible risk of radiation. In addition, it is recommended that adults designated as low caries risk (as in the present study) should have bitewing radiographs made at approximately 24-month intervals (62, 63), and the duration of the present study was 18 months.

There are several reasons why hydroxyapatite toothpastes represent an alternative to fluoride toothpastes. Hydroxyapatite is a safe active ingredient (3). Fluoride, on the other hand, can cause dental fluorosis documented also in areas of low fluoride content in the drinking water (64). It is important to note that both children and adults may ingest excessive amounts of fluoride. Children often use unappropriated toothpaste amount than recommended (65). Moreover, a recently published study indicates the negative impact of fluoride on thyroid functions in pregnant women and neuronal development in unborn (66). Therefore, reducing the overall fluoride intake is beneficial. Although those studies were often performed with populations having either water fluoridation or high fluoride concentrations in the groundwater, a risk of the chronic fluoride exposure through fluoride from fluoridated toothpastes cannot be fully excluded (i.e., there are no studies showing the safety of fluoride toothpastes in this field).

Hydroxyapatite has been shown to be not only efficient in caries prevention, but also in other fields of oral prevention, such as reduction of dentin hypersensitivity (21, 22) and improvement of periodontal health (20), biofilm control (12, 13), and whitening (24). In contrast to other active ingredients, hydroxyapatite is safe if accidentally swallowed and does not interfere with the oral microbiome and does not stain the tooth surface.

In summary, the three clinical caries studies [Paszynska et al. (6), Schlagenhauf et al. (7), and the present study] as well as a number of *in situ* (8, 12, 13, 67) studies and *in vitro* studies (9, 17, 29, 39) in the field of remineralization and biofilm control, have demonstrated that hydroxyapatite serves as an efficient and safe alternative to fluoride in anticaries toothpastes for all age groups.

## 5. Conclusion

The results of this long-term double-blinded, randomized clinical trial in adults clearly show the non-inferiority of the fluoride-free hydroxyapatite toothpaste to the toothpaste with 1,450 ppm fluoride with regard to the primary endpoint DMFS index. According to the per-protocol analysis, no increase in DMFS index was observed in 89.3% of subjects of the hydroxyapatite group and 87.4% of subjects of the fluoride group. In conclusion, hydroxyapatite was proven to be a safe and efficient anticaries agent in oral care.

## Data availability statement

The original contributions presented in the study are included in the article/supplementary material, further inquiries can be directed to the corresponding author/s.

## Ethics statement

The studies involving human participants were reviewed and approved by the Poznan University of Medical Sciences, Poznan, Poland (No. 691/20; November 4, 2020). The patients/participants provided their written informed consent to participate in this study.

## Author contributions

EP, MP, JE, and FM: conceptualization and methodology, writing—review and editing, and project administration.. TM: statistics. EP and MP: coordinating clinical investigators. EP, MP, JE, FM, AK, MG, JO-S, IK, JL-A, and ES: writing and original draft preparation. All authors contributed to the writing—review and editing, article, and approved the submitted version.
